# Co-formability, solubility enhancement and stability of olanzapine co-amorphous systems produced with different co-formers

**DOI:** 10.1080/07853890.2021.1896099

**Published:** 2021-09-28

**Authors:** Ana C. Bastos, Inês A. Santos, João F. Pinto, Ana I. Fernandes

**Affiliations:** a Centro de Investigação Interdisciplinar Egas Moniz (CiiEM), Egas Moniz Cooperativa de Ensino Superior, Caparica, Portugal; b iMed.ULisboa – Research Institute for Medicines, Faculdade de Farmácia, Universidade de Lisboa, Lisboa, Portugal

## Abstract

**Introduction:**

Strategies to address the problem of poorly water-soluble drugs encompass the conversion of a crystalline drug into an amorphous form to promote its apparent solubility and dissolution. Co-amorphous systems (CAMs) incorporate low molecular mass molecules (co-formers), which are mixed with the drug to form one single phase [1,2]. The aim of this study is to understand the capability of different co-formers (amino, carboxylic and sulphonic acids), in the production of CAMs with olanzapine by ball milling, solvent evaporation and quench cooling.

**Materials and methods:**

Mixtures (2 g) of olanzapine (OLZ) and each co-former [l-aspartate (ASP); l-tryptophan (TRY); l-arginine (ARG); l-proline (PRO); citric acid (CIT); tartaric acid (TAR); oxalic acid (OXA); saccharine (SAC); potassium acessulfame (ACE); cyclamic acid (CYC)] in 1:1 molar ratios were submitted to ball milling (BM), solvent evaporation (SE) and quench cooling (QC). CAMs were evaluated for the OLZ solubility increase, co-formability and storage stability over time, by differential scanning calorimetry (DSC), X-ray powder diffraction (XRPD) and Fourier-transform infra-red spectroscopy (FTIR).

**Results:**

The BM technique presented the most promising results followed by QC and SE (Table 1), since more CAMs were produced. All the sulphonic acids (SAC, CYC and ACE) formed CAMs regardless of the technique used, presenting complete amorphization probably due to their higher ability to produce intermolecular interactions, like hydrogen-bonding, thus increasing the stability of CAMs (more than 8 weeks at 25 °C/11 and 53% RH). Conversely, amino acids were the least efficient in producing CAMs. Crystalline OLZ presented low solubility (40.1 mg/L) in water and a general increase in the solubility of the CAMs was observed. Carboxylic acids (TAR, CIT, OXA) achieved the biggest increase (up to 269 times, BM with TART) followed by sulphonic acids (up to 199 times, SE with SAC), unveiling the possibility of improved dissolution profiles and bioavailability. **Discussion and conclusions:** The study has shown the possibility of converting a crystalline drug into an amorphous entity, particularly when in presence of co-formers which stabilise the amorphous structures formed. In fact, with sulphonic acids, both SE and BM, achieved complete amorphization and successfully stabilised the CAMs obtained. Due to the noteworthy increase in solubility, resulting from co-amorphization, this technique is considered to be adequate to process active compounds with poor water solubility, such as OLZ.

**Table 1.** Comparison of the of co-amorphization ability using different techniques and co-formers.

Mixtures	QC	SE	BM
OLZ:PRO	Yes^a^	No	No
OLZ:ARG	No	No	No
OLZ:TRY	No	No	Yes^a^
OLZ:SER	No	No	No
OLZ:ASP	No	No	Yes^a^
OLZ:CIT	Yes^a^	No	Yes^b^
OLZ:TAR	Yes^a^	No	Yes^b^
OLZ:OXA	Yes^a^	No	No
OLZ:SAC	Yes^b^	Yes^b^	Yes^b^
OLZ:CYC	No	Yes^b^	Yes^b^
OLZ:ACE	Yes^b^	Yes^b^	Yes^b^

^a^Incomplete amorphization; ^b^complete amorphization.
